# Robust AI-Driven Segmentation of Glioblastoma T1c and FLAIR MRI Series and the Low Variability of the MRIMath© Smart Manual Contouring Platform

**DOI:** 10.3390/diagnostics14111066

**Published:** 2024-05-21

**Authors:** Yassine Barhoumi, Abdul Hamid Fattah, Nidhal Bouaynaya, Fanny Moron, Jinsuh Kim, Hassan M. Fathallah-Shaykh, Rouba A. Chahine, Houman Sotoudeh

**Affiliations:** 1 MRIMath, 3473 Birchwood Lane, Birmingham, AL 35243, USA; yassine.barhoumi@mrimath.com (Y.B.); afattah@mrimath.com (A.H.F.); 2 Department of Electrical and Computer Science, Rowan University, Glassboro, NJ 08028, USA; bouaynaya@rowan.edu; 3 Department of Radiology, Baylor College of Medicine, 1 Baylor Plaza, Houston, TX 77030, USA; 4 Department of Radiology, Emory University, 100 Woodruff Circle, Atlanta, GA 30322, USA; jinsuh.kim@emory.edu; 5 Department of Neurology, University of Alabama at Birmingham, 510 20th Street South, Birmingham, AL 35294, USA; hfshaykh@uabmc.edu; 6 RTI International, Durham, NC 27709, USA; rchahine@rti.org

**Keywords:** glioblastoma multiforme, AI-based segmentation, Sørensen–Dice score, neuro-radiology, MRI imaging, sensitivity and specificity, machine learning in medical diagnosis, MRIMath©

## Abstract

Patients diagnosed with glioblastoma multiforme (GBM) continue to face a dire prognosis. Developing accurate and efficient contouring methods is crucial, as they can significantly advance both clinical practice and research. This study evaluates the AI models developed by MRIMath© for GBM T1c and fluid attenuation inversion recovery (FLAIR) images by comparing their contours to those of three neuro-radiologists using a smart manual contouring platform. The mean overall Sørensen–Dice Similarity Coefficient metric score (DSC) for the post-contrast T1 (T1c) AI was 95%, with a 95% confidence interval (CI) of 93% to 96%, closely aligning with the radiologists’ scores. For true positive T1c images, AI segmentation achieved a mean DSC of 81% compared to radiologists’ ranging from 80% to 86%. Sensitivity and specificity for T1c AI were 91.6% and 97.5%, respectively. The FLAIR AI exhibited a mean DSC of 90% with a 95% CI interval of 87% to 92%, comparable to the radiologists’ scores. It also achieved a mean DSC of 78% for true positive FLAIR slices versus radiologists’ scores of 75% to 83% and recorded a median sensitivity and specificity of 92.1% and 96.1%, respectively. The T1C and FLAIR AI models produced mean Hausdorff distances (<5 mm), volume measurements, kappa scores, and Bland–Altman differences that align closely with those measured by radiologists. Moreover, the inter-user variability between radiologists using the smart manual contouring platform was under 5% for T1c and under 10% for FLAIR images. These results underscore the MRIMath© platform’s low inter-user variability and the high accuracy of its T1c and FLAIR AI models.

## 1. Introduction

Despite recent advancements, glioblastoma multiforme (GBM), the most aggressive primary brain neoplasm, remains associated with a poor prognosis [1]. The current standard of care for GBM is maximal safe debulking, followed by concurrent chemoradiation and adjuvant chemotherapy. Magnetic resonance imaging (MRI) of the brain is the primary technique for the evaluation of treatment response and disease progression; radiologists rely on the post-contrast T1 (T1c), fluid-attenuated inversion recovery (FLAIR), T1, T2, and diffusion-weighted imaging sequences to help them detect and diagnose tumor growth [2]. It is recommended within 72 h after surgery for the assessment of residual disease, followed by subsequent MRIs every 2–6 months. Traditionally, the size of GBM on MRI is assessed by measuring the product of the maximal cross-sectional diameters [3]. However, there is evidence that 3D volumetric measurements are better at detecting the growth of low-grade gliomas (LLGs) than the maximal cross-sectional diameters, whose accuracy vs. volumetric analysis compared to previous and baseline scans is 21.0% and 56.5%, respectively [4]. Also, accurate segmentation and volumetrics are crucial for radiation planning. Finally, radiomics and texture analysis of GBM necessitate accurate segmentation of GBM on MRI. Traditionally, gliomas are segmented manually, which is time-consuming with high inter-observer and intra-observer variation [5,6]. The mean kappa score of the gross tumor volume (GTV) of newly diagnosed GBM from a Korean study was 0.58 [6]. Hence, a precise and efficient segmentation technique is needed to improve the clinical management of GBM and to answer fundamental research questions. We have developed an automated AI-based segmentation technique for segmenting brain neoplasms on different MRI sequences and a smart manual contouring platform for corrections when needed. Here, we examine the performance of AI models for T1c and FLAIR GBM sequences as compared to board-certified neuro-radiologists. We also study the inter-user variability of the MRIMath smart manual contouring software (version v1.0.0).

## 2. Methods

### 2.1. AI Generation

We have developed a proprietary training model architecture through extensive experimentation and iterative refinements. The training model features a U-Net architecture [7], designed as the backbone for end-to-end fully supervised training. Our implementation includes modifications to optimize performance for MRI data segmentation. This model incorporates inception blocks [8] to enhance feature extraction across multiple scales and employs robust initialization and regularization techniques such as dropout [9] and L2 normalization [10] to prevent overfitting. At its core, the architecture relies on an encoder–decoder structure, where the encoder progressively compresses the input into a condensed feature representation, and the decoder expands these features back to the image dimensions, aiming to predict tumor presence with high precision per pixel. The decoder then reconstructs the segmented output, culminating in precise segmentation through deterministic convolution and Softmax activation. Data augmentation techniques [11] such as rotation, flipping, translation, and Gaussian noise injection were employed to enhance the robustness of the model against variations in MRI scans. The model was trained from scratch using a proprietary dataset leveraging the TensorFlow framework [12] for defining and training the architecture. We utilized a standard training loop with an Adam optimizer [13], setting the learning rate at 0.06 and a batch size of 24. Training continued for up to 500 epochs with early stopping implemented to prevent overfitting. Data preprocessing involves converting 2D Dicom files into 2D numpy arrays [14], maintaining the patient-specific folder structure to ensure that training and validation splits are carried out per patient rather than per file. Images were resized to 256×256 pixels and normalized to have pixel values between 0 and 1 for training stability. Hyperparameters were determined iteratively, selecting the best model configuration for deployment and statistical analysis.

### 2.2. Training Data and Golden Truth

The training data study involved a comprehensive dataset comprising 2181 T1c series and 1556 FLAIR series MRI scans, featuring resolutions ranging from 256 × 256 to 512 × 512, with data collected between 2001 and 2020, acquired from various universities, community hospitals, and imaging centers across the United States. For T1c, the number of slices per series ranged from 21 to 248, averaging 94.28 slices, with a standard deviation of 75.29, while the FLAIR series had between 21 and 200 slices, averaging 31.30 slices with a standard deviation of 27.54.

MRI magnetic field strengths for T1c included 3.0 T (*n* = 1218), 1.5 T (*n* = 961), and 1.0 T (*n* = 1), with one unspecified. The FLAIR MR series used similar field strengths, with 961 scans at 3.0T, 585 at 1.5 T, and minor counts at 1.0 T (*n* = 2) and unspecified (*n* = 8). The acquisition-type distribution for FLAIR was predominantly 2D (*n* = 1433), with a smaller proportion of 3D acquisitions (*n* = 103).

Various contrast agents were employed depending on machine compatibility and specific imaging requirements, including Gadolinium, Prohance, Omniscan, Dotarem, Magnevist, Multihance, and Optimark.

All golden truth segmentation was conducted by a board-certified neuro-oncologist. The datasets were randomly divided into training (80%) and validation (20%) sets. The detailed imaging parameters for the T1C and FLAIR series are presented in Table A1, Table A2, Table A3 and Table A4.

### 2.3. Testing Dataset

#### 2.3.1. Inclusion Criteria

1. Older than 18 years of age.
2. Pathological diagnosis of glioblastoma multiforme.
3. The patient must have had an MRI of the brain with and without contrast that includes T1C and FLAIR sequences.
4. The data is anonymized.

#### 2.3.2. Exclusion Criterion

1. The T1C or FLAIR series have missing slices.

A total of 78 patients met the inclusion criteria: 17/78 were excluded because their T1C and FLAIR series had missing slices.

#### 2.3.3. Sample Size Calculation

To evaluate the accuracy, we compare the performance of the FLAIR and T1C AIs to the consensus golden truth; this procedure generates overall DSC proportions between 0 and 1. We chose to consider an overall DSC proportion of 88% as the reference value in a comparison using a two-sided, one-sample Z-proportion hypothesis testing.

We base our sample size calculation on the hypothesis that the proportion of Sørensen–Dice Similarity Coefficient scores (DSC) exceeding the designated threshold of 88% differs from 50%. We expect that our AI will exceed this threshold 70% of the time. The comparison will be made using a two-sided, one-sample Z-test and type I error alpha of 0.05. Using R version 4.1.1, specifically the pwr package for power analysis [15,16], we estimate that a sample of 42 MRIs is adequate to provide 80% power.

In our work, we are interested in testing our AI on both pre- and post-operative MRIs. We expect at least a third of the participants to have both pre- and post-operative MRI scans that can be used in our study. Therefore, we randomly selected 31 subjects from the pool of participants that meet our inclusion/exclusion criteria.

#### 2.3.4. Studies/Series Selection Procedure

Most brain tumor patients are treated at university hospitals, though some may be initially diagnosed at community hospitals. Because our intention is to produce a sample of GBM MRIs that represent the US, we gave preference to MRI studies performed at community hospitals and imaging centers. This approach resulted in 26 studies performed at university hospitals, compared to 20 studies at community hospitals and imaging centers, totaling 46 studies.

Given the prevalence of 1.5 T machines over 3.0 T machines, especially at smaller institutions, preference was given to 3 T magnets, resulting in 12 studies acquired by 3 T magnets and 34 by 1.5 T magnets. T1C 3D acquisitions, being less common than 2D, especially at community hospitals, were preferred due to their informativeness, leading to 25 series acquired in 3D and 21 in 2D. Preference was also given to FLAIR series with more than 25 slices, resulting in 28 FLAIR sequences with fewer than 25 slices and 18 sequences with more than 25 slices (range = 26–200).

The selection procedure detailed above resulted in 46 MRI studies from 31 patients, acquired from a diverse set of 19 centers across the United States, including 13 community hospitals and clinics, 4 imaging centers, and 2 university hospitals. The MRIs spanned various magnetic field strengths—1.5 Tesla (*n* = 33), 3.0 Tesla (*n* = 12), and some unspecified (*n* = 1)—and were performed using equipment from major manufacturers—GE Medical Systems (Convington, GA, USA) (*n* = 18), Philips Medical Systems (Oakwood, GA, USA) (*n* = 13), Philips Healthcare (Andover, MA, USA) (*n* = 9), and Siemens (Erlangen, Germany) (*n* = 6). The MRI acquisition types included both 2D (T1c: *n* = 21; FLAIR: *n* = 44) and 3D (T1c: *n* = 25; FLAIR: *n* = 2) formats. The imaging parameters are shown in Table A5, Table A6, Table A7 and Table A8. Various contrast agents were employed depending on machine compatibility and specific imaging requirements, including Gadolinium, Prohance, Omniscan, Dotarem, Magnevist, Multihance, and Optimark. The MRI image resolution configurations included 256 × 256, 288 × 288, 384 × 384, 432 × 432, and 512 × 512. The T1c repetition time ranged from 9.3 to 666.7 ms; the echo time ranged from 2.6 to 14.9 ms.

#### 2.3.5. Annotators, Tasks, and Golden Truth

Three board-certified neuro-radiologists annotated the testing datasets. Each neuro-radiologist was tasked with manual contouring of the T1c and FLAIR sequences of the 46 MRIs of patients diagnosed with GBM. They used the smart manual contouring platform of the MRIMath platform. Specifically, they uploaded the images onto the MRIMath platform, performed manual segmentations of the T1c and FLAIR sequences, and then downloaded the data to their computers. They shared the data with the MRIMath for analysis. A single consensus ground truth was generated from the annotations of the three neuro-radiologists by majority voting, considering each pixel as a tumor or not, based on at least 2 out of 3 votes.

### 2.4. Evaluation Metrics

#### 2.4.1. Overall Dice Score per Patient

The Dice Score (DSC) is utilized to measure the accuracy of the segmentation compared to a golden truth. It is calculated as follows:

$$\mathrm{DSC}=\frac{2\times \mathrm{TP}+\epsilon }{2\times \mathrm{TP}+\mathrm{FP}+\mathrm{FN}+\epsilon }$$

In this equation:

- TP (true positive) represents accurately segmented pixels.
- FP (false positive) indicates erroneously segmented pixels.
- FN (false negative) denotes missed pixels in the segmentation process.
- ϵ is a minor constant added for computational stability.

where ϵ is an arbitrarily chosen small constant set to $10^{-6}$. This value is used to prevent division by zero in cases where the denominator is null, and ensuring DSC equals 1, in cases where both predicted and GT values are zero, correctly reflecting a perfect match. Thus, the use of ϵ ensures numerical stability in the computation of the DSC.

True positives pertain to slices where tumors are identified and confirmed by a specified reference, serving as the ground truth (GT) for a given comparison.

#### 2.4.2. Sensitivity and Specificity

These metrics are defined as follows:

$$\begin{array}{cc} \mathrm{Specificity} & =\frac{\mathrm{True}\;\mathrm{Negatives}}{\mathrm{True}\;\mathrm{Negatives}+\mathrm{False}\;\mathrm{Positives}}\\ \mathrm{Sensitivity} & =\frac{\mathrm{True}\;\mathrm{Positives}}{\mathrm{True}\;\mathrm{Positives}+\mathrm{False}\;\mathrm{Negatives}} \end{array}$$

Sensitivity and specificity are measured both at the per-slice and per-pixel levels, the latter evaluating each individual pixel per scan.

#### 2.4.3. Hausdorff Distance

In our analysis pipeline, the Hausdorff distance quantifies the discrepancy between ground truth (GT) and predicted segmentation outcomes in the MRI T1c and FLAIR test datasets. In particular, the 95th percentile (Hausdorff 95) is used as a metric for evaluating segmentation accuracy, computed per lesion on a slice-by-slice basis within 3D scans [17,18,19].

Initially, each 3D image volume, consisting of multiple 2D slices, is identified and labeled as “independent objects” within both GT and prediction masks. For each grouped pair of objects, the Hausdorff distance was calculated at a 95% confidence interval. We report the means, standard deviations, and confidence intervals of the Hausdorff distances across the dataset.

### 2.5. Statistical Methods

To test the hypothesis that the proportion of overall AI DSC measurements exceeding the designated threshold of 88% differs from 50%, a two-sided, one-sample Z-test was utilized. Proportions of DSC above the threshold, along with their corresponding 95% confidence interval and *p*-values, are presented. An alpha level of 0.05 was employed to assess significance [15,16]. The analyses were conducted using R version 4.1.1.

Box plots were utilized to visually represent the distribution of Dice scores. To compare the volumes measured by the AI and three neuro-radiologists, the following methods were applied:

- Linear regression ($R^{2}$) for measuring the degree of correlation.
- Bland–Altman analysis was used to assess the agreement between two methods of clinical measurement. To evaluate the range within which the vast majority (95%) of the differences are expected to lie, we define the Limits of Agreement (LoAs) as the mean difference ±1.96 times the standard deviation of the differences, which represents a critical value of the standard normal distribution at a 95% confidence level. We report the 95% confidence intervals for the mean difference.
- Cohen’s Kappa Score (κ) measures the agreement between two raters who categorize instances into mutually exclusive categories [20]. The Kappa statistic for AI-based medical imaging evaluates the agreement between the AI algorithm’s segmentation and expert radiologist’s annotations, calculated as $\kappa =\frac{P_{o}-P_{e}}{1-P_{e}}$, where $P_{o}$ is the observed agreement and $P_{e}$ is the expected agreement.

To assess the statistical significance of the Kappa scores, we calculated *p*-values using a z-score method, which accounts for the variability in Kappa estimation. This involves computing the standard deviation of Kappa and the z-score to derive the *p*-value, thereby providing a robust measure of agreement significance beyond chance.

## 3. Results

### 3.1. Patients

The cohort comprised 31 patients diagnosed with GBM, including 16 females and 15 males, who underwent 46 MRI studies. Among these studies, 24 were preoperative and 22 were postoperative MRIs. The mean age of the patients was 56.68 years, with a standard deviation of 12.76 years. The median age was 58 years with an interquartile range (IQR) of 14.25 years. The racial distribution was predominantly white (*n* = 29) with African American (*n* = 2), mirroring the incidence rates in the US population (white = 83.2%, black = 5.9%) [21].

### 3.2. Hypothesis Testing

We evaluated the hypothesis that the proportion of overall AI DSC measurements exceeding a designated threshold ($p_{0}=0.88$) differs from 50%. The two-sided, one-sample Z-test revealed the following:

- For the FLAIR AI, the DSC proportions exceed $p_{0}$, 74% of the time, with a confidence interval (CI) of (60%, 84%) and a *p*-value of 0.001. This result indicates that our proportion is significantly different than 50%.
- For T1C, the DSC proportions exceed $p_{0}$, 89% of the time, with a CI of (77%, 95%) and a *p*-value of <0.001. This also implies that our proportion is significantly different than 50%.

These results demonstrate that the measurements from our device, based on both AI models, are statistically significant and clinically relevant. The high proportion of DSCs exceeding the $p_{0}$ threshold demonstrates the efficacy of the AI in aligning with the consensus ground truth, from the three radiologists. This high level of agreement underscores the high potential of our AI models to support and enhance diagnostic accuracy in clinical settings, as detailed in Table 1.

### 3.3. Dice Score Comparisons

**Figure 1:**
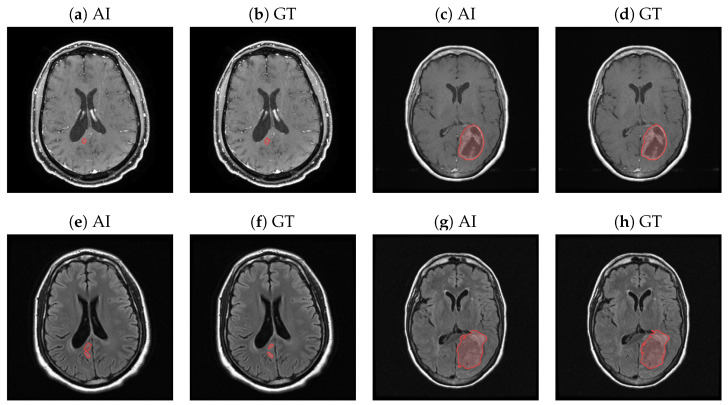
Contours of the AI (**a**,**c**,**e**,**g**) and consensus GT (**b**,**d**,**f**,**h**) for the T1c (**a**–**d**) and corresponding FLAIR series (**e**–**h**). (**e**,**f**) are the FLAIR sequences that correspond to the small tumor in (**a**,**b**). (**g**,**h**) are the FLAIR sequences that correspond to the large tumor in (**c**,**d**). Tumor segmentation is marked with a semi-transparent red overlay and delineated by a solid red outline.

In this comprehensive analysis, we present a comparative analysis of the performance of AI-generated segmentation with the consensus GT, as well as the inter-radiologist agreement for the FLAIR and T1c imaging modalities. We measure the DSC of the entire dataset and of the true positive set, i.e., the slices with tumors. Figure 1 displays the T1c and FLAIR AI-generated segmentation alongside the consensus GT delineations for small and large tumors. The depicted tumors are highlighted with a semi-transparent red overlay and are delineated by a solid red outline. The comparison clearly demonstrates a high degree of agreement between the AI-generated segmentation and the consensus GT, affirming the efficacy of both FLAIR and T1c AIs in accurately reproducing the expert radiologists’ assessments.

#### 3.3.1. Overall Dice Scores for T1c and FLAIR Modalities

Table 2 illustrates the agreement level between AI-generated segmentation and consensus GT for both the T1c and FLAIR modalities, along with the consistency of inter-radiologist assessments. The AI–consensus pairing for the T1c modality achieves a mean DSC of 94.72%, with a 95% CI of (93.31%, 96.13%), similar to the mean DSC values observed for the radiologist’s pairings ranging from 95.44% to 95.78%. For the FLAIR modality, the AI–consensus pairing achieves a mean DSC of 89.47%, with a 95% CI of (86.82% to 92.12%), comparable to those obtained from the radiologist comparisons, whose mean DSC ranges from 89.32% to 91.64%. The box plots of the overall T1c and FLAIR DSC reveal a consistent median convergence across all tested pairings, suggesting synchronized performance between the AI system and radiologists (Figure A2).

These results underscore the following:

1. The solid alignment of the T1c and FLAIR AIs with the consensus GT.
2. The low variability between the radiologists using the MRIMath Smart contouring platform.

#### 3.3.2. True Positive Dice Scores

**Figure A1:**
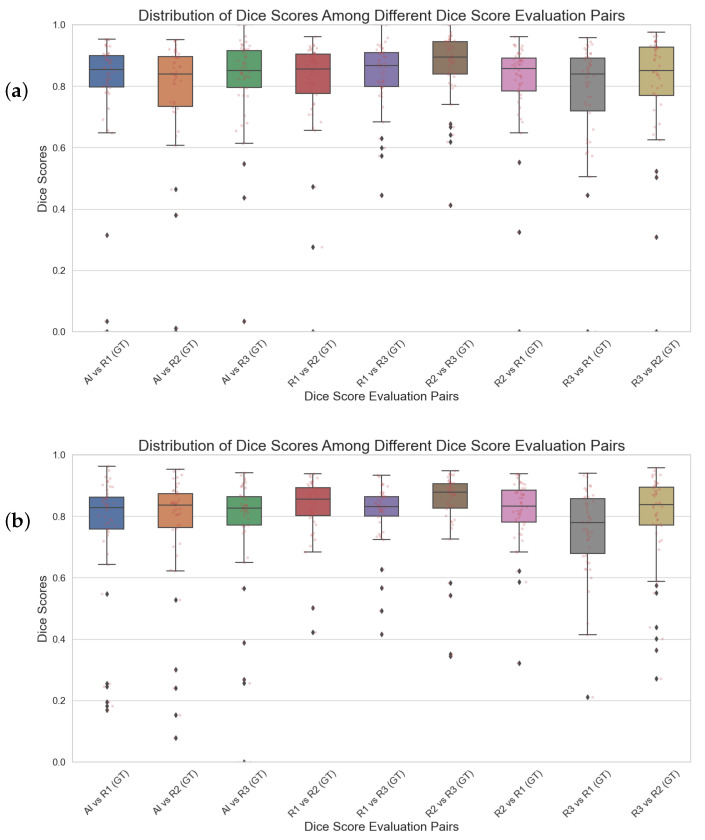
Comparison of Dice score true positive distributions for different pairings between AI and radiologists. Box plot showing the Dice score distributions for the six different combinations between AI and radiologists for (**a**) T1c, and (**b**) FLAIR modalities.

**Figure A2:**
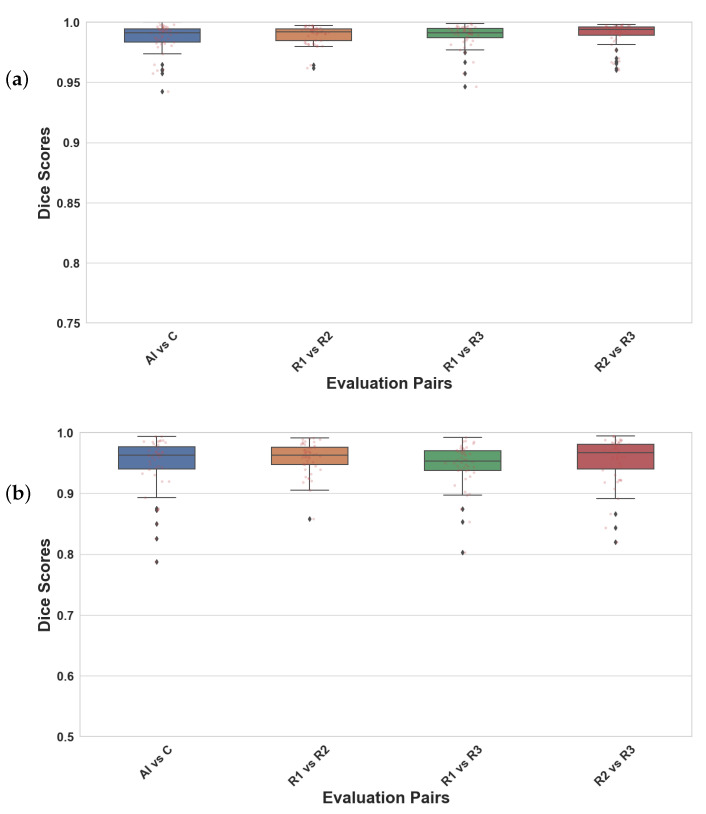
Comparison of Dice score distributions for different pairings between AI and radiologists for (**a**) T1c, and (**b**) FLAIR modalities.

The true positive DSC for both T1c and FLAIR are also similar to the measurements obtained by comparing the radiologists (see Table A13). In particular, the T1C and FLAIR AI models mean that DSCs are 81.43% and 77.62% with 95% CI ranges of (75.60%, 87.26%) and (71.42%, 83.81%), respectively. The mean DSC between radiologists ranges from 76.33% to 86.09% and 75.10% to 83.38% for T1c and FLAIR images, with a 95% CI of (70.33%, 89.42%) and (71%, 87.22%), respectively. The box plots for the true positive DSC are shown in Figure A1.

#### 3.3.3. Dice Score Subgroup Analysis

We conducted a subgroup analysis focusing on different settings within the dataset, including institutional type (university hospitals, community, and imaging centers), MRI manufacturers (GE, Philips, Siemens), lesion size, single and multiple tumors, field strength, acquisition type (2D, 3D), and operative status (pre, post). Table 3 reveals that the segmentation models exhibit a high degree of accuracy and consistency, highlighting the robustness of the models across various clinical and technical settings. The lowest DSC of 85.18% is measured from small tumors for FLAIR imaging, reflecting the increased sensitivity of the DSC to smaller tumor volumes, where minor segmentation inaccuracies become more significant.

### 3.4. Sensitivity and Specificity

This section presents an in-depth analysis of slice-wise and pixel-wise specificity and sensitivity for both T1c and FLAIR modalities, comparing AI-generated results with those obtained from the neuro-radiologists. The slice-level analysis for the T1c and FLAIR modalities show that the AI models achieve high specificity and sensitivity, closely aligning with or even surpassing radiologist benchmarks (Table A9). Specifically, the specificity metrics across all AI–radiologist pairings indicate near-perfect performance (T1c: 97.49%, FLAIR: 96.10%), suggesting a high degree of accuracy in correctly identifying negative cases. Sensitivity results are also robust (T1c: 91.63%, FLAIR: 92.09%) and comparable to the levels observed between radiologists.

At the pixel level, the specificity and sensitivity assessments, shown in Table A11 and Table A12, reveal pixel-level specificity (T1c: 99.97%, FLAIR: 99.87%) that remains consistently near-perfect across all AI-radiologist pairings. Sensitivity, although slightly lower than specificity (T1C: 89.11%, FLAIR: 86%), is within an acceptable range.

### 3.5. Hausdorff Distance

Table 4 reveals that, as compared to a radiologist, the AI exhibits a consistent range of mean Hausdorff distances, all of which are notably below 5 mm for both T1 and FLAIR modalities. This uniformity highlights the AIs’ capacity to reliably capture the essential contours of the segmented objects with a high degree of fidelity across modalities [1,3,4].

### 3.6. Volume Measurements

#### 3.6.1. Tumor Volumes: Linear Regression

**Figure 2:**
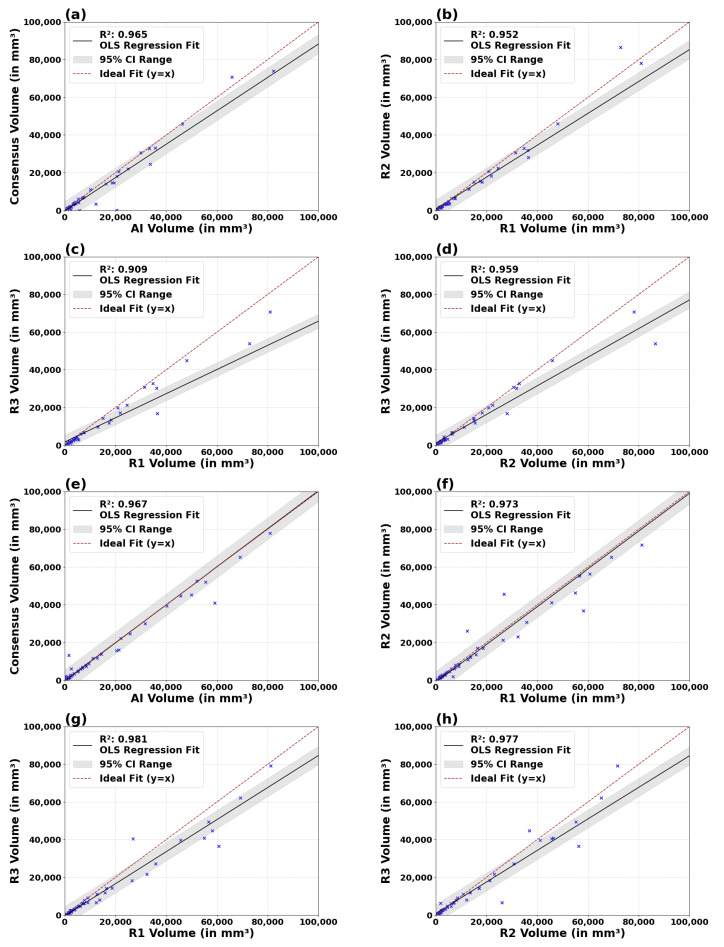
Linear regression analysis of volumes across AI and radiologist pairings for both T1C (**a**–**d**) and FLAIR (**e**–**h**) series. The analyses include AI vs. consensus GT (**a**,**e**), R1 vs. R2 (**b**,**f**), R1 vs. R3 (**c**,**g**), and R2 vs. R3 (**d**,**h**). The red dashed line represents the y = x line; the solid line represents the OLS regression line; the gray region indicates the confidence interval; the blue “x” marks denote the data points. The results of regression around the OLS line and x = y are summarized in Table A14.

Figure 2 plots the linear relationship between the volumes measured by T1C and FLAIR AI versus the consensus FT and among the radiologists. The results confirm a high degree of correlation and agreement between the consensus GT and both the T1c ($R^{2}=0.965$ for the OLS line; $R^{2}=0.939$ for the y=x line) and FLAIR AI ($R^{2}=0.967$ for both the OLS line and y=x lines). A detailed comparison across different radiologist pairings reveals that the agreement between the AI and the consensus GT $R^{2}$ is similar to the radiologists’ for both the OLS and the y=x lines (Table A14). In panels (a) and (e), the T1c and FLAIR AI models demonstrate an exceptional correlation with the consensus ground truth, as evidenced by $R^{2}$ values close to 1 and regression slopes nearly equivalent to the line y=x, indicating not only high predictive accuracy but also volume conservation in the tumor segmentation. These findings are corroborated by the high degree of alignment in the regression slopes and intercepts.

Specifically, the slopes of the best fits of the AI to consensus GT are closer to 1 (0.886 for T1c and 1,007 for FLAIR) as compared to most of the comparisons between radiologists (0.845, 0.759, 0.641 for T1c and 1.001, 0.851, 0.837 for FLAIR; Table A14). Comparative analysis among radiologists, shown in panels (b) to (d) for T1c and (f) to (h) for FLAIR, reveals a generally high level of agreement, with $R^{2}$ values consistently above 90%. Furthermore, the high $R^{2}$ among different pairings of radiologists compared to the y=x line, detailed in Table A14, reflects a high level of consistency, ensured by MRIMath©’s smart manual contouring.

#### 3.6.2. Tumor Volumes: Bland–Altman Analysis

**Figure 3:**
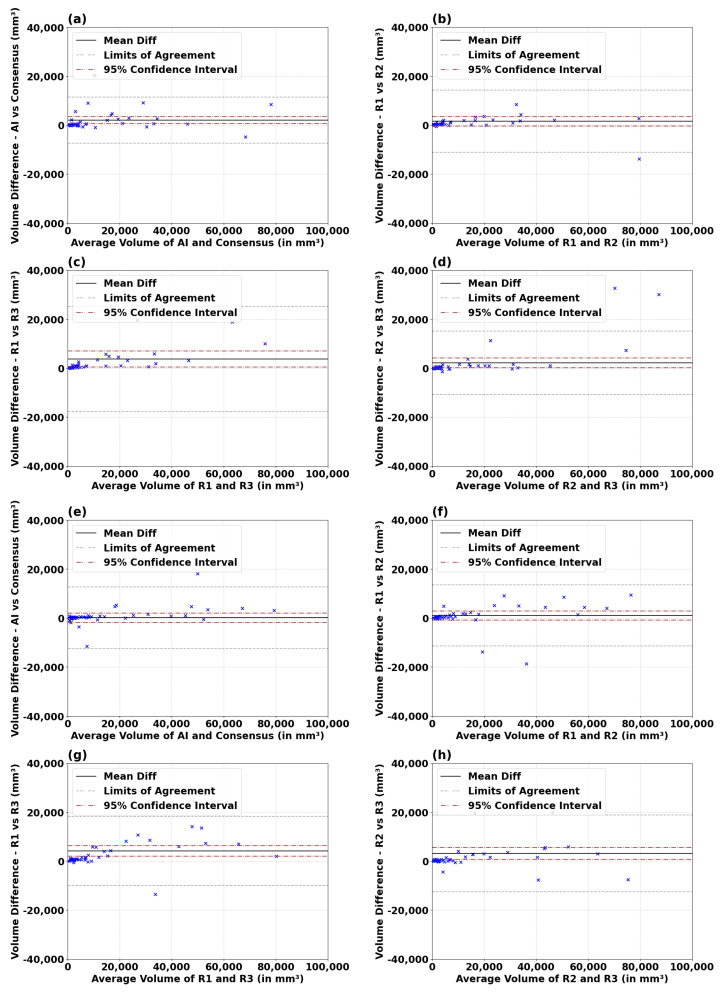
Bland–Altman analysis of volumes (in mm³) for both T1c (**a**–**d**) and FLAIR (**e**–**h**) series between AI and radiologists’ pairings. The analyses include AI vs. consensus GT (**a**,**e**), R1 vs. R2 (**b**,**f**), R1 vs. R3 (**c**,**g**), and R2 vs. R3 (**d**,**h**). Limits of Agreement (LoAs) and mean difference with 95% confidence intervals are depicted. Results are summarized in Table A15 for T1c and FLAIR modalities.

Figure 3 presents the Bland–Altman plots assessing the agreement of segmented tumor volumes between the T1C and FLAIR AIs and the consensus GT, as well as among different radiologists. The figure reveals a tight correlation between the volumes measured by the T1C and FLAIR AIs as compared to the consensus golden truth. For example, in the T1C analysis, the mean difference in volume measurement between AI and consensus GT is 2065 ${\mathrm{mm}}^{3}$ (Table A15), which is in the range of differences measured between radiologist pairings (1583 ${\mathrm{mm}}^{3},3720\;$${\mathrm{mm}}^{3}$). In the FLAIR analysis, the AI vs. consensus GT mean difference is 154 ${\mathrm{mm}}^{3}$, considerably smaller than the minimal difference between radiologists of 1040 ${\mathrm{mm}}^{3}$. Table A15 also demonstrates that the Limits of Agreement for both T1c and FLAIR AIs as compared to the consensus GT are within the range or better than what is measured from the radiologist pairings. These findings highlight AI’s capability to maintain a high level of precision and reliability in tumor volume assessments.

#### 3.6.3. Kappa Score (k)

Table A16 presents a comparative analysis of Kappa scores revealing substantial agreement with a κ scores of 0.7617 and 0.6867 for the T1c and FLAIR AIs as compared to the consensus GT, respectively. These agreement levels are within the range of scores obtained by comparing the radiologists.

### 3.7. Variability of the Smart Manual Contouring Platform of MRIMath

The results demonstrate that the manual contouring platform of MRIMath is associated with low variability (<5% for T1C and <10% for FLAIR). Furthermore, there was no statistically-significant difference between the DSCs and volumes measured by the three neuro-radiologists.

## 4. Discussion and Conclusions

Our results reveal that the T1C and FLAIR AIs’ pixel and volume predictions align closely with the manual contouring by board-certified neuro-radiologists. Furthermore, our smart manual contouring system yields low inter-user variability.

We compare the MRIMath© platform’s results side by side with other leading platforms such as Neosoma [22] and the state-of-the-art model called Swin Transformer [23], trained on the BRATS dataset [24] by Nvidia and Vanderbilt university [25]. Table 5 details key aspects of model operation, preprocessing requirements, and performance across these platforms, illustrating the unique approaches of each. MRIMath© is noted for being fully automated by not including preprocessing steps that require human supervision, like deboning, interpolation, and registration; this automation significantly simplifies the preprocessing pipeline compared to the more complex requirements of Neosoma and Nvidia’s model.

While MRIMath© processes images in 2D and treats each modality independently, Neosoma and Brats employ a 3D approach and integrate four modalities. Moreover, MRIMath© adopts a streamlined approach by handling a single series type (FLAIR or T1c), which contrasts with the subcomponent segmentations used by the other platforms. Notably, the performance of MRIMath© is highlighted with DSC scores of 89.47% for FLAIR and 94.79% for T1c. Neosoma shows an average DSC of 88.3% for preoperative data and 77.6% for postoperative data. The Nvidia model yields an average DSC of 91.3%. The output of the MRIMath© FLAIR AI is equivalent to the sum of all the subcomponents measured by Brats and Neosoma; the T1C AI segmentation is equivalent to the sum of the enhancing and necrosis subcomponents. These results underscore the distinct advantages of MRIMath© in enhancing the efficiency and accessibility of tumor segmentation for clinical applications.

As compared to the literature, the manual contouring platform of MRIMath is associated with low inter-user variability, 5% for T1C, and 10% for FLAIR. The larger inter-observer variability for FLAIR is thought to be due to vague and imprecise boundaries [5,6]. The mean kappa of the gross tumor volume (GTV) of newly diagnosed GBM from a Korean study was 0.58 as compared to 0.77 for the MRIMath manual contouring [6]. In a recent report, the mean DSC of the GTV of the FLAIR signal of low-grade gliomas was reported at 77% (substantial disagreement) [26]; in contrast, the mean DSC of the manual contouring of FLAIR images using the MRIMath smart platform was 91% (see Table 2).

The MRIMath GBM AIs can potentially by applied in radiotherapy and neurosurgical planning by improving efficiency, saving time, and lowering inter-user variability. They can also be applied to evaluate and update the current standard of care for longitudinal tumor monitoring including the RANO criteria [4]. By delivering precise and reliable segmentation within seconds, our AI tools set a foundation for accurate volumetric evaluations of tumor progression, which are pivotal for longitudinal monitoring and for clinical trials. The MRIMath smart manual contouring platform offers a safety net that allows physicians to review and approve AI segmentation efficient and with low variability. An efficient and robust segmentation is also needed for the clinical analysis of PET scans. From a research perspective, the precise segmentation capability of our AI facilitates detailed analysis of tumors, which is critical for developing predictive models in radiomics studies.

## Figures and Tables

**Table 1:** Results of the two-sided, one-sample Z-proportion test comparing the MRIMath T1C and FLAIR AIs to the reference proportion $p_{0}=0.88$.

AI Model	Proportion of DSC > $p_{0}$	Lower 95% CI	Upper 95% CI	*p*-Value
FLAIR	74%	60%	84%	0.001
T1C	89%	77%	95%	<0.001

**Table 2:** Overall DSC Statistics for T1c and FLAIR Modalities. Comparison across AI and radiologists.

Comparison	T1c		FLAIR	
	Mean	95% CI	Mean	95% CI
AI–C	94.72%	(93.31%, 96.13%)	89.47%	(86.82%, 92.12%)
R1–R2	95.74%	(94.84%, 96.65%)	91.64%	(90.13%, 93.16%)
R1–R3	95.44%	(94.25%, 96.63%)	89.32%	(87.21%, 91.43%)
R2–R3	95.78%	(94.57%, 96.99%)	90.84%	(88.59%, 93.09%)

**Table 3:** Sub-group analysis. Average Dice score for the T1c and FLAIR AIs.

Experiment Name	T1C		FLAIR	
	Mean DSC	95% C.I	Mean DSC	95% C.I
University Hospitals and Clinics	93.83%	(91.42%, 96.23%)	89.24%	(85.98%, 93.51%)
Community & Imaging Centers	95.88%	(94.61%, 97.15%)	89.10%	(84.80%, 93.41%)
Manufacturer-GE	96.60%	(95.31%, 97.89%)	88.24%	(81.80%, 93.56%)
Manufacturer-Philips	92.74%	(90.05%, 95.43%)	88.46%	(87.83%, 93.94%)
Manufacturer-Siemens	96.35%	(94.23%, 98.46%)	94.65%	(81.34%, 97.92%)
Field-1.5T	94.94%	(93.32%, 96.56%)	89.50%	(86.25%, 93.09%)
Field-3.0T	94.06%	(89.88%, 98.24%)	89.74%	(82.98%, 94.83%)
T1c Acquisition-2D	96.84%	(95.83%, 97.84%)	89.68%	(83.65%, 93.27%)
T1c Acquisition-3D	92.95%	(90.55%, 95.34%)	88.77%	(87.03%, 93.59%)
Pre-op	95.65%	(93.85%, 97.44%)	90.13%	(88.72%, 93.75%)
Post-op	93.71%	(91.31%, 96.12%)	88.15%	(82.41%, 92.68%)
Single Tumors	97.10%	(96.31%, 97.89%)	89.52%	(86.92%, 96.26%)
Multiple Tumors	91.89%	(89.24%, 94.54%)	89.04%	(85.08%, 92.00%)
Small tumors	95.79%	(93.70%, 97.88%)	85.18%	(76.51%, 89.78%)
Medium tumors	94.68%	(91.95%, 97.41%)	92.47%	(91.96%, 95.43%)
Large tumors	93.76%	(90.67%, 96.85%)	89.86%	(87.77%, 95.10%)
ALL	94.72%	(93.27%, 96.17%)	89.18%	(86.74%, 92.19%)

**Table 4:** Hausdorff 95% (in mm) for T1c and FLAIR AI. AI and radiologist Hausdorff distances.

Prediction	Ground Truth	T1c		FLAIR	
		Mean	95% CI	Mean	95% CI
AI	C	2.8943	(1.949, 4.103)	3.5217	(2.1146, 4.929)
AI	R1	3.2080	(2.182, 4.525)	4.2637	(2.5294, 5.998)
AI	R2	3.2781	(2.336, 4.602)	4.1239	(2.6128, 5.635)
AI	R3	3.1494	(2.179, 4.406)	3.9018	(2.4156, 5.743)
R1	R2	2.7666	(1.899, 3.757)	3.9871	(2.3092, 5.665)
R1	R3	2.9069	(1.765, 4.294)	4.4493	(2.4695, 6.429)
R2	R3	2.6447	(1.774, 3.756)	4.1278	(2.3834, 5.872)

**Table 5:** Comparison of preprocessing requirements, model characteristics, and performance.

Feature	MRIMath©	Neosoma [22]	Brats [25]
Deboning	Not Required	Required	Required
Interpolation	Not Required	Required	Required
Registration	Not Required	Required	Required
Data Type	2D	3D	2D/3D
Number of AIs	2	1	1
Output	1 per AI	3 Subcomponents	4 Subcomponents
Series	Single: FLAIR or T1c	Multiple: T1, T1c, FLAIR, T2	Multiple: T1, T1c, FLAIR, T2
DSC	FLAIR: 90%, T1c: 95%	Preop: 88%, Postop: 78%	Average: 90%

## Data Availability

The raw data supporting the conclusions of this article will be made available by the corresponding author on request.

## Appendix A. Dataset Demographics

### Appendix A.1. Characteristics of the Training MRI Studies

**Table A1:** Training T1c series MR imaging parameters.

Stat	Slice Thickness	Repetition Time	Echo Time	Spacing Between Slices
Mean	2.85	679.31	6.6	3.12
Std	1.65	748.61	5.63	2.28
Median	3	460.88	4.59	1.6
IQR	4	1743.24	5.89	5
Min	0.9	3.87	1.32	0.7
Max	10	9420	141	10

**Table A2:** Training T1c series MR imaging parameters—continued.

Stat	Inversion Time	Pixel Bandwidth	Echo Train Length	Imaging Frequency	Flip Angle
Mean	495.85	189.74	24.33	98.44	41.21
Std	473.97	138.11	50.11	30.7	34.56
Median	600	150	1	123.26	15
IQR	950	51.89	2	63.87	55
Min	0	61.05	0	42.59	6
Max	2550	1116.09	256	128.17	180

**Table A3:** Training FLAIR series MR imaging parameters.

Stat	Slice Thickness	Repetition Time	Echo Time	Spacing Between Slices
Mean	3.88	9459.36	141.58	3.71
Std	1.16	1589.21	51.04	2.61
Median	4	9420	141	4
IQR	2	2000	16	5.5
Min	0.47	2236.53	8.22	1
Max	5.5	15,830	422.27	7.5

**Table A4:** Training FLAIR series MR imaging parameters—continued.

Stat	Inversion Time	Pixel Bandwidth	Echo Train Length	Imaging Frequency	Flip Angle
Mean	2465.02	299.71	29.35	8,546,996.87	124.03
Std	316.36	175.53	40.25	73,023,776.54	37.24
Median	2500	287	13	123.26	90
IQR	550	82	18	59.38	80
Min	750	61.04	0	42.59	90
Max	2854.26	1302	236	639,061,410	180

### Appendix A.2. Characteristics of the Testing MRI Studies

**Table A5:** Testing T1c imaging parameters.

Stat	Slice Thickness	Repetition Time	Echo Time	Spacing Between Slices
Mean	3.13	336.14	8.71	3.71
Std	1.57	399.67	8.47	2.40
Median	3.20	113.23	7.62	3.00
IQR	3.40	599.16	5.41	4.40
Min	0.93	5.77	2.30	0.70
Max	5.00	1800.00	58.00	7.50

**Table A6:** Testing T1c imaging parameters—continued.

Stat	Pixel Bandwidth	Echo Train Length	Imaging Frequency	Flip Angle
Mean	167.69	34.78	79.59	52.41
Std	105.31	52.89	29.26	48.71
Median	161.00	1.50	63.89	30.00
IQR	52.93	99.25	44.56	81.50
Min	46.48	1.00	25.55	8
Max	559.00	122.00	127.80	180

**Table A7:** Testing FLAIR imaging parameters.

Stat	Slice Thickness	Repetition Time	Echo Time	Spacing Between Slices
Mean	4.72	9575.28	130.26	6.05
Std	0.83	1725.29	38.50	1.05
Median	5.00	9236.00	125.00	6.50
IQR	0.00	2198.00	20.38	0.50
Min	1.00	4800.00	81.00	1.00
Max	5.91	12,000.00	349.26	7.50

**Table A8:** Testing FLAIR imaging parameters—continued.

Stat	Pixel Bandwidth	Echo Train Length	Imaging Frequency	Flip Angle	Inversion Time
Mean	253.62	28.63	79.59	105.67	2475.97
Std	140.72	32.68	29.26	29.57	338.03
Median	276.00	24.00	63.89	90.00	2500.00
IQR	244.93	45.00	44.56	0.00	600.00
Min	61.05	1.00	25.55	90.00	1660.00
Max	740.00	171.00	127.80	180.00	2854.50

## Appendix B. Slice-Wise Specificity and Sensitivity

**Table A9:** T1c AI slice-level specificity and sensitivity measures.

Prediction	Specificity			Sensitivity		
	GT	Mean (%)	95% CI	GT	Mean (%)	95% CI
AI	C	97.49%	(96.08%, 98.89%)	C	91.63%	(85.21%, 98.04%)
R1	R2	99.47%	(99.15%, 99.78%)	R2	94.12%	(88.92%, 99.32%)
R2	R1	99.18%	(98.51%, 99.85%)	R1	95.05%	(90.01%, 100.08%)
R1	R3	98.53%	(97.48%, 99.58%)	R3	95.31%	(89.22%, 101.40%)
R3	R1	99.87%	(99.61%, 100.13%)	R1	91.40%	(85.12%, 97.68%)
R2	R3	98.09%	(96.86%, 99.32%)	R3	93.80%	(87.42%, 100.18%)
R3	R2	99.70%	(99.42%, 99.99%)	R2	89.81%	(83.08%, 96.54%)

**Table A10:** FLAIR AI slice-level specificity and sensitivity measures.

Prediction	Specificity			Sensitivity		
	GT	Mean (%)	95% CI	GT	Mean (%)	95% CI
AI	C	96.10%	(93.73%, 98.47%)	C	92.09%	(87.99%, 96.19%)
R1	R2	96.97%	(95.46%, 98.47%)	R2	97.92%	(96.70%, 99.13%)
R2	R1	98.02%	(96.79%, 99.25%)	R1	96.12%	(94.29%, 97.94%)
R1	R3	93.96%	(91.27%, 96.64%)	R3	98.46%	(97.12%, 99.81%)
R3	R1	98.86%	(97.97%, 99.74%)	R1	91.27%	(87.40%, 95.14%)
R2	R3	95.01%	(92.50%, 97.53%)	R3	97.95%	(96.34%, 99.56%)
R3	R2	98.92%	(98.06%, 99.77%)	R2	92.55%	(88.67%, 96.43%)

## Appendix C. Pixel-Wise Specificity and Sensitivity

**Table A11:** T1c AI pixel-level specificity and sensitivity measures.

Prediction	Specificity		Sensitivity	
	Mean (%)	95% CI	Mean (%)	95% CI
AI	99.97%	(99.96%, 99.98%)	89.11%	(82.82%, 95.40%)
R1	99.96%	(99.95%, 99.97%)	90.66%	(85.45%, 95.86%)
R2	99.99%	(99.98%, 99.99%)	78.25%	(72.68%, 83.82%)
R1	99.95%	(99.93%, 99.97%)	92.06%	(85.68%, 98.43%)
R3	100.00%	(99.99%, 100.00%)	73.37%	(67.06%, 79.68%)
R2	99.97%	(99.96%, 99.98%)	88.07%	(81.11%, 95.02%)
R3	99.99%	(99.99%, 99.99%)	81.63%	(74.85%, 88.41%)

**Table A12:** FLAIR AI pixel-level specificity and sensitivity measures.

Prediction	Specificity		Sensitivity	
	Mean (%)	95% CI (Lower, Upper)	Mean (%)	95% CI (Lower, Upper)
AI	99.87%	(99.84%, 99.90%)	86.00%	(79.05%, 92.96%)
R1	99.81%	(99.76%, 99.87%)	92.35%	(89.20%, 95.49%)
R2	99.92%	(99.88%, 99.96%)	82.96%	(79.44%, 86.49%)
R1	99.75%	(99.70%, 99.81%)	95.49%	(92.67%, 98.32%)
R3	99.96%	(99.93%, 99.99%)	74.72%	(70.46%, 78.99%)
R2	99.84%	(99.80%, 99.88%)	93.65%	(90.24%, 97.06%)
R3	99.94%	(99.90%, 99.97%)	81.74%	(77.21%, 86.27%)

## Appendix E. True Positive Dice scores

**Table A13:** TP Dice score statistics for T1c and FLAIR modalities. Comparison across AI and radiologists.

Comparison	T1c		FLAIR	
	Mean (%)	95% CI (%)	Mean (%)	95% CI (%)
AI–C	81.43	(75.60, 87.26)	77.62	(71.42, 83.81)
R1–R2	80.27	(75.23, 85.32)	82.82	(79.87, 85.78)
R2–R1	80.76	(75.96, 85.57)	81.46	(78.28, 84.65)
R1–R3	83.04	(79.87, 86.22)	80.72	(77.71, 83.72)
R3–R1	76.33	(70.33, 82.33)	75.18	(71.00, 79.37)
R2–R3	86.09	(82.77, 89.42)	83.38	(79.54, 87.22)
R3–R2	79.09	(72.75, 85.42)	78.84	(74.14, 83.53)

## Appendix F. Linear Regression Analysis

**Table A14:** Linear regression analysis of segmented volumes in mm³ for T1c and FLAIR modalities.

Modality	Comparison	Slope (a) ± Std	Intercept (b) ± Std	R^2^ (OLS)	R^2^ (x = y)
T1c	AI vs. Consensus	0.886 ± 0.051	−368 ± 1203	0.965	0.939
	R1 vs. R2	0.845 ± 0.057	751 ± 1512	0.952	0.916
	R1 vs. R3	0.641 ± 0.062	1705 ± 1620	0.909	0.579
	R2 vs. R3	0.759 ± 0.048	1115 ± 1094	0.959	0.848
FLAIR	AI vs. Consensus	1.007 ± 0.056	−327 ± 1934	0.967	0.967
	R1 vs. R2	1.001 ± 0.051	−1060 ± 1914	0.973	0.972
	R1 vs. R3	0.851 ± 0.036	−438 ± 1347	0.981	0.934
	R2 vs. R3	0.837 ± 0.039	793 ± 1492	0.977	0.930

## Appendix G. Bland–Altman

**Table A15:** Bland–Altman analysis of segmented volumes in mm³ for T1c and FLAIR modalities.

Comparison	T1c			FLAIR		
	Mean Difference	95% CI	LoA	Mean Difference	95% CI	LoA
AI vs. C	2065	(634, 3496)	(−7378, 11,508)	154	(−1758, 2067)	(−12,469, 12,777)
R1 vs. R2	1583	(−347, 3513)	(−11,155, 14,321)	1040	(−851, 2932)	(−11,441, 13,522)
R1 vs. R3	3720	(460, 6979)	(−17,791, 25,231)	4222	(2081, 6364)	(−9912, 18,356)
R2 vs. R3	2136	(166, 4107)	(−10,869, 15,142)	3182	(805, 5559)	(−12,505, 18,868)

## Appendix H. Kappa Scores

**Table A16:** Comparative analysis of Kappa scores for T1c and FLAIR modalities.

Modality	Method 1	Method 2	Kappa	Kappa Std	95% CI
T1c	AI	C	0.7617	0.0750	(0.6146, 0.9087)
	R2	R3	0.8938	0.0556	(0.7849, 1.0027)
	R1	R2	0.7943	0.0734	(0.6505, 0.9382)
	R1	R3	0.7602	0.0761	(0.6110, 0.9094)
FLAIR	AI	C	0.6867	0.0752	(0.5394, 0.8341)
	R1	R2	0.6388	0.0758	(0.4902, 0.7874)
	R2	R3	0.6314	0.0772	(0.4800, 0.7827)
	R1	R3	0.5285	0.0818	(0.3681, 0.6889)

## References

[1] Tan A.C., Ashley D.M., Lopez G.Y., Malinzak M., Friedman H.S., Khasraw M. (2020). Management of glioblastoma: State of the art and future directions. CA Cancer J. Clin..

[2] Mohammed Y.M., El Garouani S., Jellouli I. (2023). A survey of methods for brain tumor segmentation-based MRI images. J. Comput. Des. Eng..

[3] Wen P.Y., van den Bent M., Youssef G., Cloughesy T.F., Ellingson B.M., Weller M., Galanis E., Barboriak D.P., de Groot J., Gilbert M.R. (2023). RANO 2.0: Update to the Response Assessment in Neuro-Oncology Criteria for High- and Low-Grade Gliomas in Adults. J. Clin. Oncol..

[4] Raman F., Mullen A., Byrd M., Bae S., Kim J., Sotoudeh H., Moron F.E., Fathallah-Shaykh H.M. (2023). Evaluation of RANO Criteria for the Assessment of Tumor Progression for Lower-Grade Gliomas. Cancers.

[5] Vos M.J., Uitdehaag B.M., Barkhof F., Heimans J.J., Baayen H.C., Boogerd W., Castelijns J.A., Elkhuizen P.H., Postma T.J. (2003). Interobserver variability in the radiological assessment of response to chemotherapy in glioma. Neurology.

[6] Wee C.W., Sung W., Kang H.C., Cho K.H., Han T.J., Jeong B.K., Jeong J.U., Kim H., Kim I.A., Kim J.H. (2015). Evaluation of variability in target volume delineation for newly diagnosed glioblastoma: A multi-institutional study from the Korean Radiation Oncology Group. Radiat. Oncol..

[7] Ronneberger O., Fischer P., Brox T. (2015). U-net: Convolutional networks for biomedical image segmentation. Proceedings of the International Conference on Medical Image Computing and Computer-Assisted Intervention.

[8] Szegedy C., Liu W., Jia Y., Sermanet P., Reed S., Anguelov D., Erhan D., Vanhoucke V., Rabinovich A. Going deeper with convolutions. Proceedings of the IEEE Conference on Computer Vision and Pattern Recognition.

[9] Srivastava N., Hinton G., Krizhevsky A., Sutskever I., Salakhutdinov R. (2014). Dropout: A simple way to prevent neural networks from overfitting. J. Mach. Learn. Res..

[10] Zou H., Hastie T. (2005). Regularization and variable selection via the elastic net. J. R. Stat. Soc. Ser. (Stat. Methodol.).

[11] Shorten C., Khoshgoftaar T.M. (2019). A survey on Image Data Augmentation for Deep Learning. J. Big Data.

[12] Abadi M., Agarwal A., Barham P., Brevdo E., Chen Z., Citro C., Corrado G.S., Davis A., Dean J., Devin M. (2016). TensorFlow: Large-Scale Machine Learning on Heterogeneous Distributed Systems. TensorFlow White Paper.

[13] Kingma D.P., Ba J. (2014). Adam: A Method for Stochastic Optimization. Presented at the International Conference on Learning Representations (ICLR). https://arxiv.org/abs/1412.6980.

[14] Braga L., Semelka R.C., Pietrobon R., Martin D.R. (2018). Comparison of Normalization Techniques for the Intelligent Segmentation of Multimodal Medical Imaging Data. Radiology.

[15] Bulus M., Polat C. (2023). pwrss R paketi ile istatistiksel guc analizi [Statistical power analysis with pwrss R package]. Ahi Evran Univ. Kirsehir Egit. Fak. Derg..

[16] Bulus M. pwrss: Statistical Power and Sample Size Calculation Tools. R Package Version 0.3.1. https://CRAN.R-project.org/package=pwrss.

[17] Zhang Y., Zhong P., Jie D., Wu J., Zeng S., Chu J., Liu Y., Wu E.X., Tang X. (2021). Brain Tumor Segmentation From Multi-Modal MR Images via Ensembling UNets. Front. Radiol..

[18] Jia Z., Zhu H., Zhu J., Ma P. (2023). Two-Branch network for brain tumor segmentation using attention mechanism and super-resolution reconstruction. Comput. Biol. Med..

[19] Celaya A., Riviere B. (2024). A Generalized Surface Loss for Reducing the Hausdorff Distance in Medical Imaging Segmentation. arXiv.

[20] McHugh M.L. (2012). Interrater reliability: The kappa statistic. Biochem. Med..

[21] Bohn A., Braley A., Rodriguez de la Vega P., Zevallos J.C., Barengo N.C. (2018). The association between race and survival in glioblastoma patients in the US: A retrospective cohort study. PLoS ONE.

[22] Abayazeed A.H., Abbassy A., Müeller M., Hill M., Qayati M., Mohamed S., Mekhaimar M., Raymond C., Dubey P., Nael K. (2022). NS-HGlio: A Generalizable and Repeatable HGG Segmentation and Volumetric Measurement AI Algorithm for the Longitudinal MRI Assessment to Inform RANO in Trials and Clinics. Neuro-Oncol. Adv..

[23] Liu Z., Lin Y., Cao Y., Hu H., Wei Y., Zhang Z., Lin S., Guo B. Swin Transformer: Hierarchical Vision Transformer Using Shifted Windows. Proceedings of the IEEE/CVF International Conference on Computer Vision (ICCV).

[24] Menze B.H., Jakab A., Bauer S., Kalpathy-Cramer J., Farahani K., Kirby J., Burren Y., Porz N., Slotboom J., Wiest R. (2015). The Multimodal Brain Tumor Image Segmentation Benchmark (BRATS). IEEE Trans. Med. Imaging.

[25] Hatamizadeh A., Nath V., Tang Y., Yang D., Roth H.R., Xu D. (2021). Swin UNETR: Swin Transformers for Semantic Segmentation of Brain Tumors in MRI Images. Proceedings of the International MICCAI Brainlesion Workshop.

[26] Boer A.H., van der Weide H.L., Bongers E.M., Coremans I.E.M., Eekers D.B., de Groot C., van der Heide H., Niel C., van de Sande M.A.E., Smeenk R.J. (2020). Inter-Observer Variation In Tumor Volume Delineation Of Low Grade Gliomas, A Multi-Institutional Contouring Study. Int. J. Radiat. Oncol. Biol. Phys..

